# Supplementary material for: Effect of a Polypropylene Separator with a Thin Electrospun Ceramic/Polymer Coating on the Thermal and Electrochemical Properties of Lithium-Ion Batteries
Source: Polymers (Basel). 2024 Sep 17;16(18):2627. doi: 10.3390/polym16182627 (PMC11436061; doi:10.3390/polym16182627)
Supplement: Supplementary file 1 [file polymers-16-02627-s001.zip › polymers-3173462-supplementary.pdf]

Supporting Information:

Effect of a polypropylene separator with a thin electrospun ceramic/polymer coating on the thermal and electrochemical properties of lithium-ion batteries

Yeongsu Hwang[1], Minjae Kim[1,*]

1: Mechanical & Control Engineering, Handong Global University, Pohang 37554, Republic of Korea.

*: Corresponding author: mj.kim@handong.edu

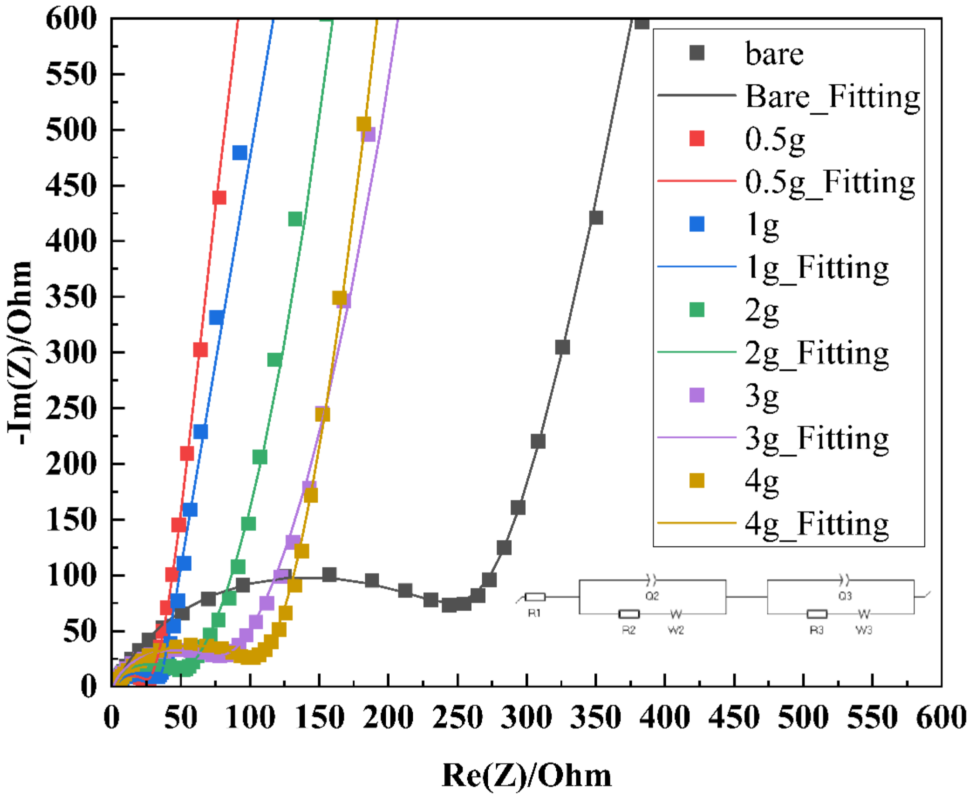

Figure S1: EIS analysis with equivalent circuit model and Nyquist plots of experimental (symbols) and fitted results (lines).
